# Membrane-bound myosin IC drives the chiral rotation of the gliding actin filament around its longitudinal axis

**DOI:** 10.1038/s41598-023-47125-5

**Published:** 2023-11-14

**Authors:** Yusei Sato, Kohei Yoshimura, Kyohei Matsuda, Takeshi Haraguchi, Akisato Marumo, Masahiko Yamagishi, Suguru Sato, Kohji Ito, Junichiro Yajima

**Affiliations:** 1 Department of Life Sciences, Graduate School of Arts and Sciences, The University of Tokyo, 3-8-1 Komaba, Meguro-ku, Tokyo, 153-8902 Japan; 2 Department of Biology, Graduate School of Science, Chiba University, 1-33, Inage, Chiba, Japan; 3 Komaba Institute for Science, The University of Tokyo, 3-8-1, Komaba, Meguro-ku, Tokyo, 153-8902 Japan; 4 Research Center for Complex Systems Biology, The University of Tokyo, 3-8-1, Komaba, Meguro-ku, Tokyo, 153-8902 Japan

**Keywords:** Motility, Cytoskeleton

## Abstract

Myosin IC, a single-headed member of the myosin I family, specifically interacts with anionic phosphatidylinositol 4,5-bisphosphate (PI[4,5]P_2_) in the cell membrane via the pleckstrin homology domain located in the myosin IC tail. Myosin IC is widely expressed and physically links the cell membrane to the actin cytoskeleton; it plays various roles in membrane-associated physiological processes, including establishing cellular chirality, lipid transportation, and mechanosensing. In this study, we evaluated the motility of full-length myosin IC of *Drosophila melanogaster* via the three-dimensional tracking of quantum dots bound to actin filaments that glided over a membrane-bound myosin IC-coated surface. The results revealed that myosin IC drove a left-handed rotational motion in the gliding actin filament around its longitudinal axis, indicating that myosin IC generated a torque perpendicular to the gliding direction of the actin filament. The quantification of the rotational motion of actin filaments on fluid membranes containing different PI(4,5)P_2_ concentrations revealed that the rotational pitch was longer at lower PI(4,5)P_2_ concentrations. These results suggest that the torque generated by membrane-bound myosin IC molecules can be modulated based on the phospholipid composition of the cell membrane.

## Introduction

Myosin IC is a single-headed, membrane-bound, actin-based motor protein that is widely expressed in both invertebrates and vertebrates^1^. Myosin IC comprises an N-terminal motor domain, a neck region containing a light chain-binding domain (LCBD), and a relatively short tail domain^2^. The motor domain, which hydrolyzes adenosine triphosphate (ATP), interacts with the actin filament to generate mechanical force. The LCBD of myosin IC contains three isoleucine–glutamine motifs that bind to calmodulins and calmodulin-like proteins and function as a mechanical lever arm^2^. The tail domain contains the pleckstrin homology (PH) domain that specifically interacts with phosphatidylinositol 4,5-bisphosphate (PI[4,5]P_2_) in the cell membrane. PI(4,5)P_2_ and its derivatives are considered minor cell membrane components and function as signaling molecules in various pathways^3^. Myosin IC physically connects and generates forces between the cortical actin filaments and membrane lipids^4,5^. Furthermore, it plays different roles in various physiological processes, including cargo transportation^4,6^, lipid raft trafficking^7^, and mechanosensing in the stereocilia of the inner ear^8^. Myosin IC and myosin ID, another member of the myosin I family, play key roles in establishing the left–right axis in the bilateria*.* In *Drosophila melanogaster*, myosin IC overexpression modifies the cell shape chirality and alters the left–right axis of organs^9,10^. Recently, a detailed kinetics analysis of *Drosophila* myosin IC was performed^11^; however, the relationship between the motor function-associated mechanical properties of membrane-bound myosin IC and its physiological role remains unclear.

The cell membrane, which separates the cytoplasm from the extracellular environment, also serves as a two-dimensional reaction interface for membrane proteins and signaling molecules for several functions, including cell migration and cell–cell interactions^12^. Previous in vitro studies have investigated the motor properties of myosin family members associated with supported lipid bilayers (SLBs)^13–15^, a widely used cell membrane model system^16,17^. Although membrane fluidity may decrease the transfer of the force generated by motor proteins to cytoskeletal filaments, motor proteins anchored to the SLBs can move cytoskeletal filaments^13,18–21^. Regarding myosin IC, actin filament gliding has been observed in SLBs containing physiological concentrations of PI(4,5)P_2_^14,15,22^. Furthermore, myosin IC-induced asymmetric motility of actin filaments, where gliding actin filaments turn in leftward circles, was increased on SLBs^14^. Further investigation of motility resulting from the interaction of membrane-bound myosin IC and actin filaments is needed to understand the biophysical properties of myosin IC and its physiological functions in the cell.

Some myosins, such as myosin II, V, VI, and X, exhibit unique mechanical properties and can generate a directional force along (on-axis) and perpendicular to (off-axis) the longitudinal axis of the actin filament. The off-axis force generated by myosin II (heavy meromyosin) has been observed via supercoiling formation caused by the rotational motion of a gliding actin filament around its longitudinal axis, with its leading part selectively fixed to the glass substrate^23^. This torque component of the cross-bridge force was also indicated by detecting the orientation of individual fluorophores sparsely labeled to the actin filament, which was free to glide over a surface coated with myosin II^24,25^ or V^25^. The helical motion of myosin V, VI, or X around the freely suspended actin filament was observed through defocused imaging^26,27^, polarized light imaging^28^, or Parallax, a technique for three-dimensional (3D) nanometer-scale tracking^29,30^. These in vitro experiments were only performed using myosins anchored to rigid surfaces, such as glass coverslips, microbeads, or quantum dots (QDs). However, the effects on torque generation of myosin motors anchored to a fluid lipid bilayer closer to the intracellular environment have not yet been thoroughly examined.

Herein, we investigated if unconventional, lipid membrane-bound single-headed myosin IC molecules could generate torque to rotate the gliding actin filaments along their longitudinal axes. We performed in vitro gliding assays on SLBs using full-length myosin IC molecules of *Drosophila melanogaster* (*Drosophila* myosin IC) and observed the displacement of QDs bound to actin filaments using a three-dimensional prismatic optical tracking (*tPOT*) microscope^31,32^. We report that a rotational motion is induced by myosin IC anchored to PI(4,5)P_2_-containing SLBs in gliding actin filaments around their longitudinal axes, a motion we termed corkscrewing. Furthermore, we assessed the gliding velocity and pitch of the corkscrewing motion based on varying PI(4,5)P_2_ concentrations in SLBs to determine the involvement of a lipid composition-dependent regulatory mechanism for torque generation by myosin IC. Additionally, we discussed the possible underlying mechanisms of torque generation by myosin IC molecules and their intracellular functions.

## Result

### SLB-bound myosin IC drove the left-handed corkscrewing motion of actin filaments

**Figure 1:**
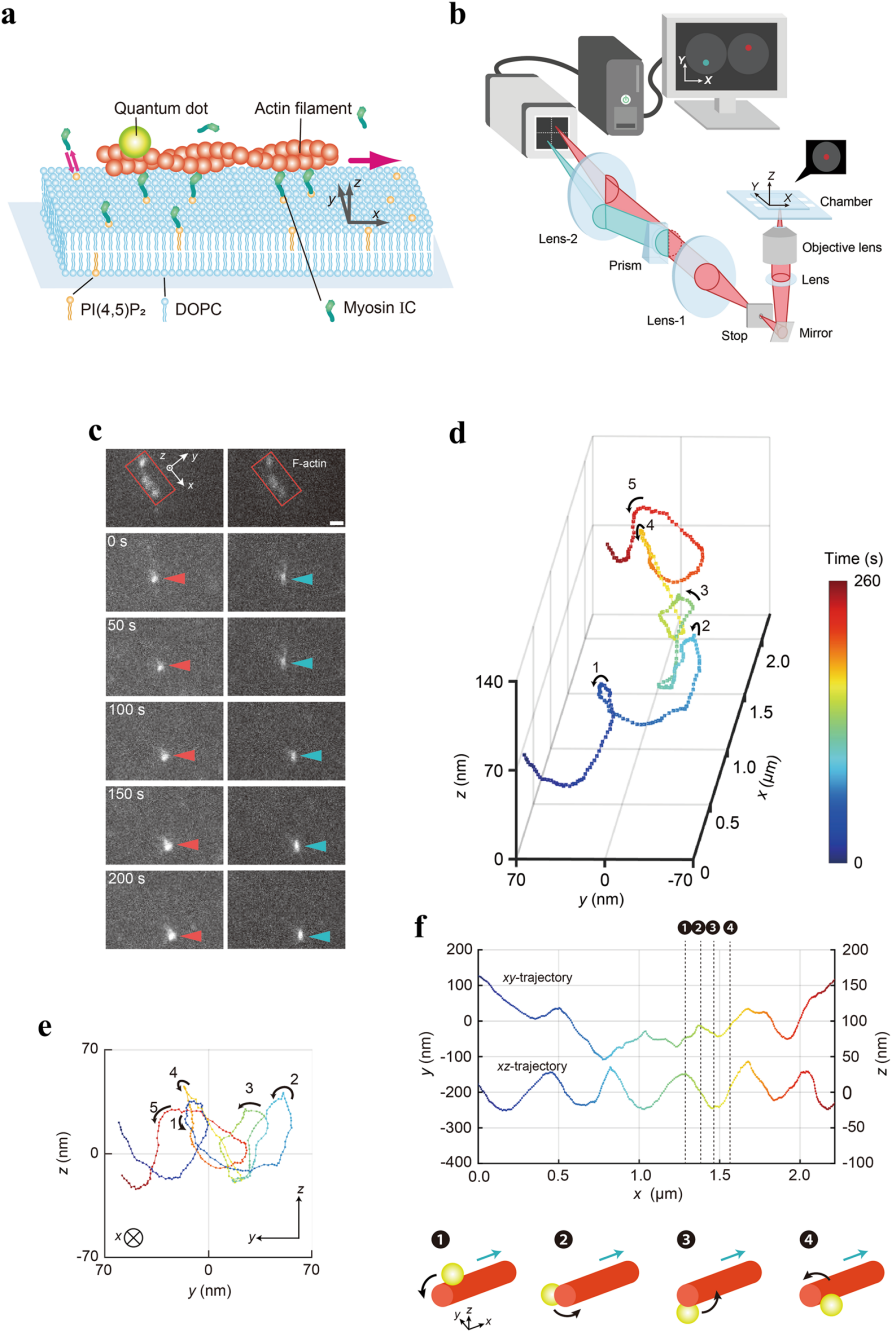
3D observation of the corkscrewing motion of myosin IC-driven gliding actin filaments. (**a**) Schematic of the in vitro corkscrewing assay. Cover glass surface is coated with PI(4,5)P_2_-containing SLBs_._ Sparsely biotinylated and Alex647-labeled actin filaments bound to QDs (*λ* = 525 nm) are driven by the PI(4,5)P_2_-bound myosin IC via the PH domain. (**b**) Schematic of the *tPOT* microscope (not to scale). The *x*- and *y*-positions of actin filament-bound QDs were obtained from the image pairs split by the prism. The *z*-position of the QD was obtained from the relative *y*-position of the split image pair. (**c**) Split image pairs captured by the *tPOT* microscope. The top panel illustrates a split image pair of a biotinylated-Alex647-labeled actin filament (marked by red rectangles). The other panels illustrate the time series of paired images of a QD bound to the actin filament gliding on the myosin IC-coated surface (pairs of red and cyan arrowheads). Scale bar = 1 µm; interval between panels = 50 s. (**d**) The 3D plot of QD revealing the left-handed corkscrewing motion of the myosin IC-driven actin filament. The color indicates the observation time (see color bar). (**e**) The *yz*-trajectory of the QD shown in **d**, the counterclockwise rotation of the actin filament gliding in the forward translocation direction. (**f**) The *xy*- and *xz*-trajectories of the QD bound to the actin filament shown in **d**. Rotation handedness is confirmed by referring to the *xy*- and *xz*-trajectories of the QD because the sinusoidal oscillation phases of the two traces shift with a quarter of the wavelength as the gliding actin filaments rotate around their longitudinal axis. The numbered labels ❶–❹ and thin black dotted lines indicate the peaks and midpoints for a given period of the sinusoidal-like *xz*-trajectory. Schematic of the left-handed corkscrewing motion of the actin filament over time (bottom). Red cylinder and yellow sphere represent the actin filament and QD, respectively. The blue and black curved arrows indicate the gliding and rotation directions of the actin filament, respectively. The numbers ❶–❹ correspond to those indicated above. Optimally, the *z*- and *y*-coordinates of the QD bound to the actin filament have maximum values at ❶ and ❷ and minimum values at ❸ and ❹, respectively. In case actin filaments are gliding along a curved path, the *y*-coordinate accordingly shifts.

To gain insights into the motility of myosin IC anchored to a diffusive lipid environment, we examined the 3D motion of a QD bound to a gliding actin filament driven by SLB-bound full-length *Drosophila* myosin IC. SLBs are highly viscous fluids and can function as a substrate that transmits the myosin-generated force to drive the gliding of actin filaments^13–15,18^. SLBs are formed by fusing small unilamellar vesicles (SUVs) comprising the neutral phospholipid 1,2-dioleoyl*-sn-*glycero-3-phosphocholine (DOPC) with the anionic phospholipid PI(4,5)P_2_ on hydrophilic glass coverslips. We used SLBs containing 6% PI(4,5)P_2_ and 94% DOPC for the standard gliding assay. To assess the lipid fluidity of SLBs, fluorescence recovery after photobleaching (FRAP) experiments were performed (Supplementary Fig. 1a). The diffusion coefficient of the lipids in SLBs (*D*_LIPID_) with 0.05% (mol/mol) ATTO647N–1,2-dioleoyl-*sn*-glycero-3-phosphoethanolamine (DOPE) as the fluorescent tracer was 2.7 ± 0.4 µm^2^ s^−1^ (*n* = 5, 4 independent SLBs; Supplementary Fig. 1b and Supplementary Table 1) by fitting the fluorescence recovery curve^14^; this was consistent with the values reported in previous studies^14,19,33,34^. Furthermore, we prepared SLBs containing 1% (mol/mol) 1,2-distearoyl-*sn*-glycero-3-phosphoethanolamine–N-(biotinyl[polyethyleneglycol]-2000) (DSPE–PEG-2000-biotin) in a flow chamber and added fluorescent streptavidin (Avi-488) to the flow chamber so that Avi-488 was anchored to DSPE–PEG-2000-biotin. To measure the diffusivity of Avi-488 in the SLBs, individual Avi-488 molecules were tracked using a total internal reflection fluorescence (TIRF) microscope to obtain the single-molecule trajectories (Supplementary Fig. 1c). The ensemble average diffusion coefficient of Avi-488 (*D*_Avi_) was 1.1 ± 0.3 µm^2^ s^−1^ (*n* = 56 tracked molecules, 11 independent experiments; Supplementary Fig. 1d and Supplementary Table 1) by fitting the mean square displacement as a function of time; the result was consistent with that of a previous study (a diffusion coefficient of 1.5 ± 0.1 µm^2^ s^−1^ for Avi-488)^19^. The obtained values indicate that the fluidity of PI(4,5)P_2_-containing SLBs, which interact with the PH domain of the myosin IC tail, is sufficient to facilitate the lateral diffusion of myosin IC on the SLB surface in the corkscrewing assay (Fig. 1a).

To determine the *xyz*-positions of streptavidin-coated QDs bound to sparsely biotinylated actin filaments gliding across the PI(4,5)P_2_-containing SLB-bound myosin IC-coated surface, we used a *tPOT* microscope (Fig. 1a–c). The *tPOT* microscope, originally developed to track single fluorescent molecules^35^, QDs^31^, microbeads^36^, or cells^37^, achieved nanometer-order accuracy in the 3D positioning of actin filament-bound QDs fixed on a glass coverslip^38^ (Supplementary Fig. 2). Using this method, we observed that the 3D trajectory of the QDs bound to the gliding actin filaments exhibited helical motion (Fig. 1d, Supplementary Fig. 3a, and Supplementary Movie 1); this indicates that myosin IC causes the rotation of gliding actin filaments around their longitudinal axis (corkscrewing motion). These corkscrewing motions have not been previously observed for myosin I family members and demonstrate the torsional and axial gliding force production characteristics of single-headed myosin IC. The 3D trajectory (Fig. 1d) and *yz*-trajectory (Fig. 1e) of the SLB-bound myosin IC-driven QD-tagged actin filament revealed the left-handed direction of rotation of the actin filament. Furthermore, the *xy*- and *xz*-trajectories of the QDs showed oscillations; however, the *xy*-oscillatory trajectories of the QDs were often irregular, indicating that the QD-bound gliding actin filaments often bent and drastically changed their gliding direction (Fig. 1f and Supplementary Fig. 3b). A phase lag was often observed between the sinusoidal oscillations of the *xy*- and *xz*-trajectories, with the *xy*-trajectory lagging slightly behind the *xz*-trajectory; this also indicates the left-handed corkscrewing motion of the actin filament (Fig. 1f). The torsional force by myosin IC that caused the rotation of the gliding actin filaments was effective even when bound to the diffusive lipid membrane. The handedness of the myosin IC-driven corkscrewing motion in the actin filaments was consistent with those reported in a previous study on dimeric myosin II and V^25^.

### Quantification of corkscrewing actin filaments driven by membrane-bound myosin IC

**Figure 2:**
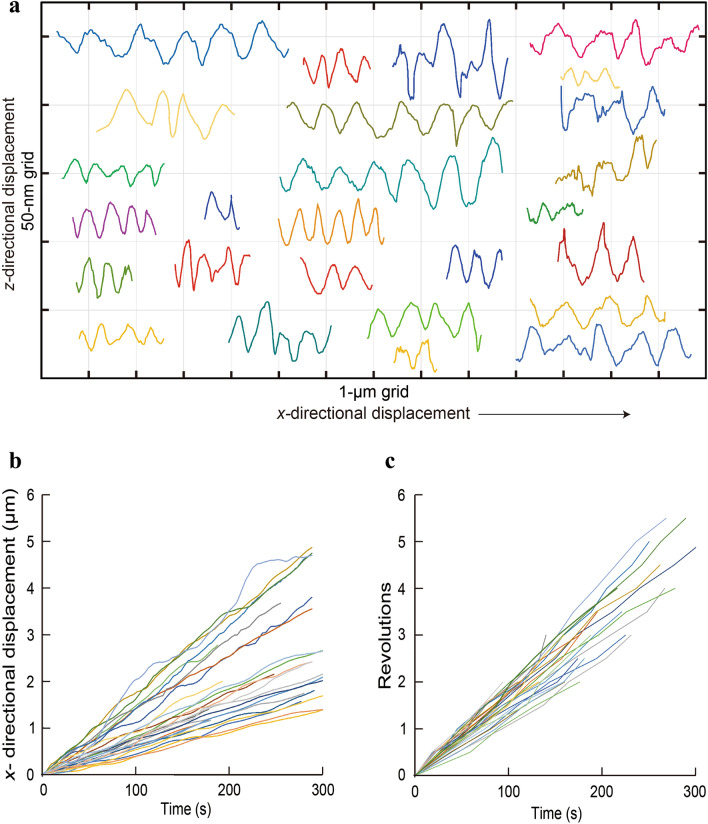
Individual traces of corkscrewing actin filaments on SLBs. (**a**) *xz*-trajectories of the QDs bound to corkscrewing actin filaments. Each *xz*-trajectory of the QDs (0.72 µM myosin IC, 6% PI(4,5)P_2_, *n* = 26) exhibited a robust corkscrewing motion with a relatively constant pitch. Individual traces of the QDs in (**a**) were fitted with a sinusoidal function to obtain the corkscrewing pitch. Time courses of the *x*-displacements (**b**) and revolutions (**c**) of the QDs bound to corkscrewing actin filaments. Individual traces of the QDs in **b** and** c** were fitted with linear functions to obtain the gliding and rotational velocities, respectively. Average gliding and rotational velocities were 9.5 ± 4.0 μm s^−1^ and + 0.016 ± 0.003 rev s^−1^, respectively.

In the in vitro actin filament corkscrewing assay, myosin IC-driven gliding actin filaments often changed their direction of motion in the *xy*-plane owing to their flexibility. Thus, the *xy*-trajectories of the QDs bound to gliding actin filaments also changed accordingly. In contrast, most of the *xz*-trajectories of the QDs exhibited constant oscillations (Figs. 1f and 2a). We selected the actin filament-bound QDs that glided reasonably straight and further analyzed their 3D positions. From the *xz*-trajectories that showed ≥ 2 oscillations, the corkscrewing pitches (that is the gliding distance of the actin filament per rotation around its longitudinal axis) of each actin filament were determined by fitting the *xz*-trajectory of the QD with sinusoidal function (Fig. 2a). The mean corkscrewing pitch of myosin IC was 0.58 ± 0.16 μm (mean ± standard deviation, SD) under 6% PI(4,5)P_2_-containing SLBs (Supplementary Table 2). This corkscrewing pitch was markedly longer than the intrinsic helical pitch of the actin filament (approximately 72 nm), suggesting that the helical trajectories of QDs occurred owing to the intrinsic properties of myosin IC. The gliding and rotational velocities of the corkscrewing motion were determined by linear approximation of the plots of *x*-displacements (Fig. 2b) and revolutions (Fig. 2c) versus time for individual QDs with ≥ 2 revolutions. The mean gliding and rotational velocities were 9.5 ± 4.0 nm s^−1^ (mean ± SD) and + 0.016 ± 0.003 rev s^−1^ (mean ± SD, the plus ( +) sign represents left-handed revolution), respectively (Supplementary Table 2).

### Effect of PI(4,5)P_2_ concentration in SLBs on the corkscrewing motion

**Figure 3:**
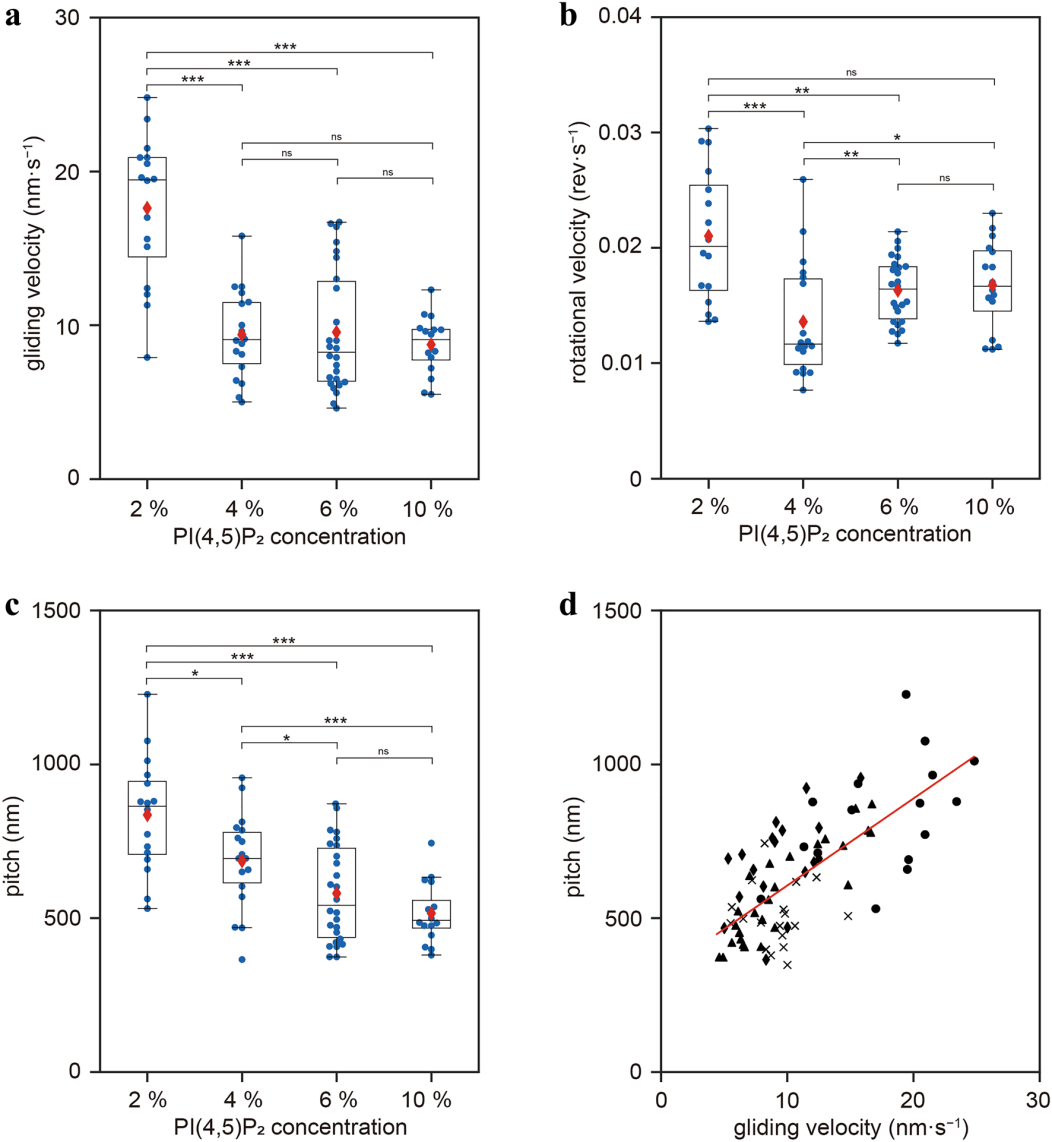
Dependence of the corkscrewing motion on PI(4,5)P_2_ concentration. Gliding velocity (**a**), rotational velocity (**b**), and pitch (**c**) versus PI(4,5)P_2_ concentration of individual QDs bound to corkscrewing actin filaments at an applied myosin IC concentration of 0.72 µM. The box represents the 75th–25th percentiles, and the median and mean values are indicated by black lines and red diamonds, respectively [*n* = 16 (2%), 18 (4%), 26 (6%), and 18 (10%)]. ***,* p* < 0.001; **, *p* < 0.01; *, *p* < 0.05; and ns, nonsignificant in the Wilcoxson rank-sum test. (**d**) Correlation of the gliding velocity and pitch of the corkscrewing motion on SLBs containing 2% (black filled circle), 4% (black filled rhombus), 6% (black filled triangle), and 10% ( ×) PI(4,5)P_2_. Red line indicates a linear approximation of the plots. The correlation coefficient between the gliding velocity and corkscrew pitch was 0.71 (*n* = 78).

The PH domain in the myosin IC tail has a high affinity for the anionic phospholipid PI(4,5)P_2_^3^. Although relatively low, LCBD also has an affinity for anionic phospholipids such as PI(4,5)P_2_ and phosphatidylserine^3,39^. In a previous study, the gliding velocity of myosin IC-driven actin filaments on fluid SLBs in an in vitro gliding assay decreased with increasing concentrations of anionic phospholipids in SLB^14^. It has been suggested that the gliding velocity may be affected by the inhibition of motor domain dynamics caused by the interaction of lever arm-containing LCBD with anionic phospholipids in SLB. To investigate the effect of anionic phospholipids in SLBs on torque generation, we performed the corkscrewing assay using SLBs with varying PI(4,5)P_2_ concentrations (2–10 mol%) and *Drosophila* myosin IC. The gliding velocity of actin filaments at 2% PI(4,5)P_2_ concentration was faster than that at 4–10% PI(4,5)P_2_ concentrations (Fig. 3a); this was consistent with the finding of a previous study on mouse myosin IC^14^. The mean gliding velocity at 2% PI(4,5)P_2_ concentration was approximately twice that at 10% PI(4,5)P_2_ concentration; no significant differences in gliding velocity were observed at 4%, 6%, and 10% PI(4,5)P_2_ concentration (Supplementary Table 2). The rotational velocity was the slowest at 4% PI(4,5)P_2_ concentration and approximately two-thirds of the fastest velocity at 2% PI(4,5)P_2_ concentration (Fig. 3b and Supplementary Table 2). Moreover, we examined the effect of myosin IC concentration on gliding velocity and found that gliding velocity was not affected by myosin IC concentration (Supplementary Fig. 4). Furthermore, we found that the corkscrewing pitch was sensitive to changes in PI(4,5)P_2_ concentrations; the pitch at low PI(4,5)P_2_ concentration was longer than that at high PI(4,5)P_2_ concentration (Fig. 3c and Supplementary Table 2). The plots of gliding velocity versus corkscrewing pitch at all PI(4,5)P_2_ concentrations were approximated linearly, indicating a strong correlation (correlation coefficient = 0.71) between them within the range of the experimental conditions (Fig. 3d). Because the corkscrewing pitch value was determined by dividing gliding velocity by rotational velocity, the longest pitch value at 2% PI(4,5)P_2_ concentration indicates that the ratio of gliding velocity to rotational velocity is the highest. A change in the corkscrewing pitch implies a change in the ratio of gliding to rotational velocities; therefore, PI(4,5)P_2_ concentrations in SLBs may have different effects on the torsional and longitudinal forces generated by myosin IC.

## Discussion

To investigate the motility of myosin IC on SLBs mimicking the cell membrane, 3D nanometry using the *tPOT* microscope was applied to quantify the movement of actin filaments driven by full-length, single-headed *Drosophila* myosin IC anchored to the PI(4,5)P_2_-containing fluidic SLBs. The QDs bound to gliding actin filaments were tracked in 3D and the corkscrewing motions of actin filaments driven by nonprocessive myosin IC were observed. The direction of the off-axis torque generated by SLB-bound myosin IC was consistent with that reported for myosin II, V, and X anchored to solid substrates such as glass coverslips, microbeads, and QDs^25,30^. Our present experimental system requires the binding of QDs to actin filaments as tracers; therefore, we cannot rule out the possibility that the presence of QDs bound to actin filaments promotes or inhibits the myosin IC-driven corkscrewing motion. Quantifying corkscrewing motion using a single fluorescent dye molecule^25,35^ as a tracer instead of QD will help elucidate the effect of QDs on the corkscrewing motion.

The mechanisms underlying the filament rotation by myosins remain unclear; however, several factors have been proposed to contribute to the corkscrewing motion of cytoskeletal filaments by nonprocessive motor proteins, including the off-axis component of motor domain conformational change, motor filament interaction-induced molecular drag, or directionally biased selection of the motor to the filament binding site^25,40–42^; it can be inferred that the corkscrewing motion is the result of a combination of different mechanisms. Moreover, it has been proposed that the rotational motion of processive myosins, such as myosin V and VI, results from the interaction between the stride length of myosin and the helical disposition of actin monomers^26,27^. Based on this approach, the stride length of processive myosin slightly differs from that of the actin helical structure, resulting in helical motions of processive myosins. However, nonprocessive myosins, such as myosin IC and skeletal muscle myosin II, have short stride lengths; thus, the abovementioned mechanism that factors the stride length is insufficient to clearly explain the observed corkscrewing pitch that reaches the submicrometer level (Fig. 2a)^24,25^. If myosin IC followed the monomers on the filaments, the corkscrewing pitch should match the pitch of the actin helical structure (approximately 72 nm); however, our study revealed that the corkscrewing pitch by monomeric myosin IC on SLB was approximately 0.5 µm, suggesting the involvement of a mechanism of corkscrewing motion that is independent of myosin stride length.

A previous theoretical study explained the corkscrewing motion of actin filaments gliding across a surface covered with multiple nonprocessive myosin monomers. In the model, myosin monomers were anchored to the cover glass and preferentially bound to the approaching specific actin monomers within the filaments, inducing the rotation of gliding actin filaments^43^. The actin monomers, termed “target zone,” to which myosin motor domains selectively bind, are periodically located in the filament^44,45^. The left-handed corkscrew motion of the actin filament around its longitudinal axis arises from the selective binding of myosin motor domains to the target zone located to the left of the longitudinal axis of the filament, pulling in the direction of the long axis of the filament, that is, rotating the filament around the longitudinal axis during the stroke of the lever arm along the longitudinal axis of the filament. Herein, the handedness of the myosin IC-induced corkscrewing motion (Fig. 1d) is consistent with that of the rotation estimated by the aforementioned model. Furthermore, the model predicts that the stiffness of the myosin motor domain affects the magnitude of the torque and corkscrewing pitch, as greater stiffness pulls the target zone more strongly^43^. It is likely that at high PI(4,5)P_2_ concentrations in fluidic SLBs, the mechanical properties of the lever arm, which weakly interacts with PI(4,5)P_2_^3^, are altered, resulting in a shorter corkscrewing pitch (Fig. 3c). The selectivity of myosin heads regarding actin monomers is also predicted to be affected by the gliding velocity of the actin filament^43^. A strong correlation was observed between the gliding velocity and corkscrewing pitch (Fig. 3d), suggesting that the corkscrewing pitch by myosin IC can exhibit sensitivity toward their gliding velocity. Herein, the results indicate that the corkscrewing motion can be explained by the selective binding of myosin motor domains to target zone actin filaments, although the possibility of oblique power strokes of the lever arms cannot be ruled out.

We demonstrated the chiral corkscrewing motion of gliding actin filament driven by SLB-bound myosin IC, containing varying concentrations of PI(4,5)P_2_, indicating that the lipid composition of the membrane, a factor not previously considered, may regulate torque generation by myosin IC. A possible mechanism by which the PI(4,5)P_2_-containing membrane may directly regulate the myosin IC-generated torque is the electrostatic interaction of PI(4,5)P_2_ with LCBD^14^, which acts as a mechanical lever arm. If the interaction between PI(4,5)P_2_ and LCBD inhibits conformational changes in the motor domain during the mechanochemical cycle and alters the ratio of the translational force component to the rotational force component in response to PI(4,5)P_2_ concentration (Fig. 3a,b), the corkscrew pitch can be altered (Fig. 3c).

Although the mechanism of torque generation by myosin IC remains unclear, the torque component in the motor domain may be efficient for myosin IC to transport vesicles around obstacles in a crowded intracellular environment^46^, similar to that proposed for other myosin^30^ and microtubule-based motor proteins, such as kinesins and dyneins^47,48^. In addition to being an intracellular transporter, myosin IC functions as an antagonist of myosin ID, another member of the myosin I family, during the chirality formation of the epidermal cells of *Drosophila melanogaster* organs. Various studies have shown that myosin IC overexpression results in an inversion of the organ phenotype, and the knockdown of myosin IC results in left–right asymmetry defects in the organ^9,49^. In particular, whole-body twisting induced by myosin IC overexpression in the larval epidermal tissue, which is devoid of left–right asymmetry, implies that myosin IC plays a crucial role in establishing the left–right asymmetry in organisms^15^. Myosin IC is responsible for regulating the effect of the myosin ID-induced left–right asymmetry of *Drosophila*; the motor domain determines organ chirality^15^. There are notable differences in myosin IC and ID motilities because myosin ID propels the actin filaments in leftward circles (circular motion) in the in vitro gliding assay, whereas myosin IC does not^15^. Furthermore, vesicles coated with multiple myosin IC and ID molecules can move processively along actin filaments, with myosin ID having a longer run length than myosin IC^11^. However, the mechanism by which these motile properties contribute to the establishment of cell and organ chirality remains unknown. Although the rotation of gliding actin filaments around their longitudinal axis (corkscrewing motion) driven by myosin IC observed in the present study does not directly address the question of the molecular mechanism underlying the circular motion of the actin filament, myosin IC, which exhibits the left-handed corkscrewing motion (Fig. 1), should be a left-to-right force component in the direction perpendicular to the longitudinal axis of actin filaments; in contrast, myosin ID, which exhibits a circular left-rotational motion^15^, should be a right-to-left force component. Considering that the actin-attachment lifetime of myosin IC is ninefold longer than that of myosin ID^11^, myosin IC may suppress the circular left-rotational motion of myosin ID-driven actin filaments and be involved in establishing left–right asymmetry in cells and organs. Quantifying the direction of the myosin ID-induced corkscrewing motion may provide further insights into the underlying molecular mechanism.

Myosin IC may participate in establishing cell chirality, along with cadherin and β-catenin, which are responsible for cell–cell adhesion^10^. Myosin IC, which binds to the actin filaments longer than myosin ID, may serve as a tether that holds actin filaments to the cell membrane^11^. Formin, a factor that promotes actin polymerization is also essential for establishing left–right asymmetry in *Drosophila*^50^. Formin drives the left-handed rotation of actin filaments around their longitudinal axis while processively polymerizing actin filaments^51^. It has been speculated that the rotation of polymerizing actin filament by formin induces the formation of counterclockwise cell chirality^52^. The torque generated by formin may be transmitted to the cell membrane via myosin IC bound to the actin filaments, resulting in cell chirality^11^. Because the torque of the left-handed corkscrewing motion produced by myosin IC is in the same direction as that exerted by formin on the actin filaments, the two torque components may reinforce each other. Furthermore, the tension in the actin filament network near the cell membrane may be altered by the torque exerted by myosin IC molecules tethered to the actin filaments, which may be the direct role of the torque generated by myosin IC in this process.

Reportedly, myosin IC directly regulates the cell morphology in neuronal growth cone^53^. Asymmetric inhibition of myosin IC in the growth cone causes the rearrangement of the actin cytoskeleton network and changes the cell shape in the direction of the inhibition area. Torque generation around the longitudinal axis of actin filaments generated by myosin V^54^ and X^55^, which interact with the cell membrane and actin cytoskeleton, has suggested to be involved in the formation of chiral-shaped filopodia. Considering that myosin IC is bound to the cell membrane via its tail domain and to the cortical actin cytoskeleton via its motor domain, it is conceivable that the torque force generated by myosin IC at the cell membrane surface may also be involved in regulating cell membrane morphology and cortical actin cytoskeleton organization. Future studies with higher spatial resolution observations of the in vivo 3D dynamics of the cortical actin filament and myosin IC are necessary to reveal the biological role of torque generation by myosin motor proteins.

## Materials and methods

### Construction of plasmids for myosin IC and calmodulin

The *Drosophila melanogaster* myosin IC gene was inserted into the pFastBac vector (Invitrogen, Carlsbad, CA, USA) to generate the pFastBac *Drosophila* myosin IC baculovirus transfer vector. Myosin IC (UniProt ID: Q23979) cDNA cloned from *Drosophila melanogaster* (a gift from Dr. K. Matsuno) was mutated to create an NcoI site in the upstream region of the nucleotide sequence encoding residue 1 and an AgeI site in the downstream region of the nucleotide sequence encoding residue 1035 of myosin IC. This DNA was cut with NcoI and AgeI and the fragment was ligated with the NcoI-AgeI fragment of the pFastBac XI-K Full vector^56^. The resulting construct, *Drosophila* full-length myosin IC (pFastBac *Drosophila* myosin IC), encodes a FLAG-tag (DYKDDDDK) in the N-terminus and a Myc tag (EQKLISEEDL) and His8-tag (HHHHHHHH) in the C-terminus. The *Drosophila melanogaster* calmodulin gene was inserted into the pFastBac vector to generate the pFastBacCaM baculovirus transfer vector. The calmodulin cDNA cloned from *Drosophila melanogaster* (a gift from Dr. K. Matsuno) was mutated to create an XbaI site upstream of the nucleotide sequence encoding residue 1 and an XhoI site downstream of the nucleotide sequence encoding 149 residues of *Drosophila* calmodulin. The BamHI-KpnI digest fragment was ligated with the BamHI-KpnI digest fragment of pFastBac. DH10Bac was transformed with pFastBacMyo1c and pFastBacCaM, respectively, to obtain the bacmid of baculovirus. Transfection of the bacmid into SF9 resulted in baculoviruses with myosin IC and calmodulin constructs.

### Preparation of biological samples

*Drosophila* myosin IC was co-expressed with *Drosophila* calmodulin in High Five™ cell culture (Invitrogen). Cells were harvested and washed with 10 mM HEPES, pH 8.0, 150 mM NaCl, and 1 mM MgCl_2_. The pelleted cells were suspended with 3 vol/g cells of buffer A (30 mM HEPES, pH 8.0, 200 mM NaCl, 50 mM KCl, 5 mM MgCl_2_, 10% glycerol, 10 mM β-mercaptoethanol, 0.5 µM *Chara corallina* calmodulin, and a mixture of protease inhibitors) containing 7 mM ATP. Then, 3 vol/g cells of buffer A containing 1% Nonidet P-40 was added, and the ingredients were mixed. *Chara* calmodulin, which was added to supplement *Drosophila* calmodulin during purification, has 88% amino acid identity with *Drosophila* calmodulin. After incubation on ice for 15 min, the lysate was centrifuged at 228,000×*g* for 30 min at 4 °C. The supernatant was mixed with 0.3 mL of nickel-nitrilotriacetic acid-agarose (Qiagen) in a 50-mL tube on a rotating wheel for 1 h at 4 °C. ﻿The resin suspension was then loaded on a column and washed with 30 mL of buffer A containing 30 mM imidazole, followed by 10 mL of buffer B (30 mM HEPES, pH 8.0, 50 mM KCl, 5 mM MgCl_2_, 30 mM imidazole, 10% glycerol, 10 mM β-mercaptoethanol, approximately 0.5 µM *Chara corallina* calmodulin, and a mixture of protease inhibitors) containing 2 mM ATP. *Drosophila* myosin IC was eluted with buffer B containing 250 mM imidazole. The eluted *Drosophila* myosin IC was ﻿mixed with 0.3 mL of anti-FLAG M2 affinity resin (Sigma-Aldrich) in a 50-mL tube on a rotating wheel for 1 h at 4 °C. The resin suspension was then loaded on a column and washed with 10 mL of buffer C (25 mM HEPES, pH 7.4, 150 mM KCl, 4 mM MgCl_2_, 10% glycerol, 0.5 mM DTT, approximately 0.5 µM *Chara corallina* calmodulin, and a mixture of protease inhibitors) containing 2 mM ATP, followed by washing with 30 mL of buffer D (25 mM HEPES, pH 7.4, 25 mM KCl, 4 mM MgCl_2_, 10% glycerol, 0.5 mM DTT, *Chara corallina* calmodulin, and a mixture of protease inhibitors) containing 0.2 mM ATP. *Drosophila* myosin IC was eluted with buffer D containing 0.2 mg/mL FLAG peptide (Sigma-Aldrich). The purity and homogeneity of protein samples were confirmed by SDS–polyacrylamide gel electrophoresis (SDS-PAGE) (Supplementary Fig. 5). *Chara corallina* calmodulin (Q9LDQ9) was expressed in *E. coli* strain BL21 (DE3) and purified using the trichloroacetic acid method and affinity chromatography using Phenyl Sepharose CL-4B^57^. Purified myosin IC and calmodulin were rapidly frozen in liquid nitrogen and stored in a −80 °C freezer. G-actin was purified by a standardized protocol from acetone powder prepared from rabbit skeletal muscle using a previously described method^58^. G-actin was polymerized at a total actin concentration of 1.2 μM in F buffer (10 mM HEPES–KOH, pH 7.5, 100 mM KCl, 1 mM ATP, 1 mM MgCl_2_) and stabilized with 4 µM Alexa 647-labeled phalloidin (Invitrogen). Then, actin filaments were labeled with 1 mM Biotin-(AC5)_2_ Sulfo-OSu (Dojindo, Kumamoto, Japan) in the buffer (10 mM HEPES–KOH, pH 7.5, 0.2 mM ATP, 0.2 mM CaCl_2_, 0.2 mM DTT) for 1 h at 4 °C. The actin filament mixture was ultracentrifuged, and excess biotin was removed by washing with F buffer before suspension. The precipitate of biotinylated actin filaments was suspended in F buffer.

### SLB preparation

Glass coverslips (24 mm × 36 mm, 130–170 μm thickness; Matsunami Glass Ind., Japan) were washed with a 5 M KOH solution and then rinsed with ultrapure water. The coverslips were dried and plasma cleaned (PDC-32D; Harrick Plasma) for 30 min. The flow chamber was assembled with 24 mm × 36 mm and 18 mm × 18 mm glass coverslips using two thin strips of tape (810-1-18, Scotch 3 M) that were placed approximately 3 mm apart. The volume of the flow chamber was approximately 5 µL. Giant unilamellar vesicles (GUVs) were prepared via the inverted emulsion method^59^. The lipid-in-oil mixture was prepared by dissolving the desired phospholipids in medium-chain triglyceride oil (Nisshin OilliO Group, Japan) by heating the mixture at 60 °C for 3 h in the water bath. For the corkscrewing assay, the lipid-in-oil mixture contained 90–98% DOPC (molecular weight 786, Avanti Polar Lipids) and 2–10% PI(4,5)P_2_ (molecular weight 1096, Avanti Polar Lipids). For FRAP and Avi-488 single-molecule tracking, the lipid-in-oil mixture contained 0.05% ATTO647N–DOPE (molecular weight 855, ATTO-TEC GmbH) and 1% DSPE–PEG-2000-biotin (molecular weight 3016, Avanti Polar Lipids). To form water-in-oil droplets, the lipid-in-oil mixture was suspended in an inner solution (20 mM HEPES–KOH, pH 7.5, 75 mM NaCl, 80 mM sucrose, 300 mM glucose). The emulsion was gently loaded onto the outer solution (20 mM HEPES–KOH, pH 7.5, 75 mM NaCl, 380 mM glucose). To form the GUVs, the solutions were centrifuged for 15 min, the supernatant was removed, and the precipitated GUVs were suspended. SUV dispersions were prepared by sonicating the GUV solution for 1 h. CaCl_2_ was added to the SUV dispersion to a final concentration of 2.5 mM to facilitate SLB formation immediately before infusion into the glass flow chamber. The SUV dispersions were then added to the chambers and incubated for 30 min. The chamber was then washed with 10 volumes of the H20S75 buffer (20 mM HEPES–KOH, pH 7.5, 75 mM KCl) before use.

### FRAP experiment

The FRAP experiment was performed by opening/closing the path of a 488 nm wavelength laser for bleaching while observing SLBs with a 632 nm wavelength laser. The 632 nm wavelength laser (05-LHP-991, Melles Griot) was used at 10 mW laser power to observe ATTO647N–DOPE in SLBs, and the 488 nm wavelength laser (488 LS FP, Coherent OBIS) was used at 40 mW laser power for bleaching. The FRAP experiment was performed as follows: the prebleached SLB was recorded for 2 s and then bleached for 5 s with a 488 nm wavelength laser, followed by the recovery of the intensity of the recorded SLB. Images were acquired with a 100 ms exposure for 600 frames using a modified TIRF microscope (IX71, Olympus) fitted with a UPlan-Apochromat TIRF 100×/1.45 NA objective lens and an electron-multiplying (EM)-charge-coupled device (CCD) camera (iXon DV887DCS, Andor Technology) using the Solis software (Andor Technology). The *D*_LIPID_ value was calculated by fitting the recovery curve of the ATTO647N–DOPE fluorescence intensity^60^.

### Three-dimensional Prismatic Optical Tracking (*tPOT*) microscopy

The principle of 3D tracking has been described previously^31,61^. The back-focal-plane (BFP) of the objective was focused outside the camera port of a modified inverted microscope (Eclipse Ti-PFS, Nikon) with an achromatic lens-1 to create an equivalent BFP (eBFP) (Fig. 1b). A custom-made wedge prism (3.5°) coated with an antireflective layer was precisely positioned at the eBFP to split beam path. Two split images of the sample were focused on the camera with another lens-2, which was used to determine the magnification of the optical system. The *tPOT* microscope was equipped with a 100×/1.49 NA, plan-apochromat TIRF objective lens (Nikon), either a Cy5 filter set (Semrock) for Alexa 647 labeled actin filaments or a GFP-3035C filter set (Semrock) for QDs, a stable stage (KS-N, ChuukoushaSeisakujo, Tokyo, Japan), a LED light source (D-LEDI, Nikon), a pulse motor (SGSP-13ACT, Sigma Koki, Tokyo, Japan), and controller (QT-CM2, Chuo Precision Ind., Tokyo, Japan). Images were acquired with 500 or 1000 ms exposure for 600 or 300 frames by an EM-CCD camera (iXon X_3_ DU897, Andor Technology) via Solis software (Andor Technology). The position of the two optically separated images of a QD was determined using the 2D Gaussian fitting as (*x*1, *y*1) and (*x*2, *y*2), and *x*, *y*, and *z* were calculated using (*x*1 +  *x*2)/2, (*y*1 + *y*2)/2 and *y*1 − *y*2, respectively, using Igor software. To calibrate the real *z*-axis position, the objective was moved vertically at a constant speed using the stage equipped with the pulse motor and controller while observing the QDs bound to the glass surface. The calculated and real *z*-axis position (as defined by the pulse motor) corresponded linearly over a range of ±0.4 µm from the focal plane. The temperature of the sample stage was controlled and maintained at 25 °C using a custom-built water-circulating temperature control system (F-12, Julabo, Germany)^62^. The temperature was measured using a micro thermos sensor (HD-1100E, Anritsu Meter, Japan).

### Actin filament corkscrewing assays

The corkscrewing assays were performed using QD-labeled actin filaments. QD-labeled actin filaments were prepared by adding 50 nM streptavidin-coated QDs (Qdot525 streptavidin conjugate, Thermo Fisher Scientific) diluted in KMg25 buffer (10 mM MOPS, pH 7.0, 25 mM KCl, 1 mM DTT, 1 mM EGTA, and 1 mM MgCl_2_), mixed with an equal volume of 800 nM sparsely biotinylated and Alexa Fluor 647 phalloidin-labeled actin filament, and incubated for 30 min at room temperature. After incubation, the QD-labeled actin filament solution was diluted 1/50th of the original concentration with the KMg25 buffer. QD-labeled actin filaments were prepared for each day of the assay. Before the assay, 2 mg/mL BSA was infused into the SLB chamber and incubated for a minimum of 30 min for blocking. QD-labeled actin filaments (0.5 µL) were mixed with 1 μL of 3.25 μM myosin IC in a total of 4.5 μL of KMg25 buffer containing 1 mM ATP, 9.7 μM *Chara* calmodulin, 3 mg/mL glucose, 50 U/mL glucose oxidase (Sigma-Aldrich), 40 μg/mL catalase (Sigma-Aldrich), 0.3 mg/mL creatine kinase (Roche), and 5 mM creatin phosphate (Roche) immediately before each experiment to prepare the assay mixture. Into the SLB-coated glass flow chamber, one chamber volume of the assay mixture was infused and the chamber was then sealed using vacuum grease. In the preliminary screening for a condition that allows us to observe the stable motion of the actin filaments, we realized that high myosin concentrations should be introduced into the flow chamber. Therefore, we used a sufficient myosin concentration (0.72 µM in the flow chamber) that allowed a stable gliding movement, although we were not aware of exactly how many myosins simultaneously interact with an actin filament at any given time. The assays were performed at 25 °C. The number of QDs bound to the actin filaments was one in most cases; if more than one QD was bound, further analysis was not performed. The actin filaments were introduced into the flow chamber at a concentration such that approximately one actin filament was observed in one field of view (a circle with a radius of 7.8 μm); the mean length of the QD-bound actin filament was 2.1 ± 1.5 μm (mean ± SD, *n* = 102) (Supplementary Fig. 6). However, the actual length of the actin filament in the corkscrewing motion remains unknown because our optical system does not allow the simultaneous observation of actin filaments and QDs.

### Quantification of the corkscrewing motion of actin filaments

The acquired images of the QDs were averaged every 4 frames using ImageJ software. Based on the averaged sequential images, fluctuating (rapid forward or backward movement relative to the direction of travel), wiggling (frequent small movements to the left or right relative to the direction of travel), or crossing QDs were ignored with no further analysis. Using Igor software, we tracked the QDs bound to actin filaments that exhibited stable directional movement. For further analysis, *x*-, *y*-*,* and *z*-positions in the individual trajectories were averaged every 20 frames. The 3D trajectories were displayed using the MATLAB software, which has an easy-to-use graphical display, to determine if the trajectories exhibited the corkscrewing motion (Fig. 1d and Supplementary Fig. 3). Among the 17, 21, 32, and 22 QD movements for 2%, 4%, 6%, and 10% PI(4,5)P_2_ concentrations, respectively, that were analyzed to obtain the 3D trajectories, cases that were not suitable for further analyses were observed; in these cases, the QDs did not exhibit a clear corkscrewing motion [*n* = 1, 2% PI(4,5)P_2_; *n* = 2, 4% PI(4,5)P_2_; *n* = 3, 6% PI(4,5)P_2_; and *n* = 3, 10% PI(4,5)P_2_] or QDs exhibited frequent stops (*n* = 1, 4% PI(4,5)P_2_; *n* = 1, 6% PI(4,5)P_2_); and *n* = 1, 10% PI(4,5)P_2_]. Furthermore, the amplitude of projection was too small owing to some unknown reason (< 25 nm, half as large as the sum of the radii of the actin filament and QD, *n* = 2, 6% PI(4,5)P_2_). Finally, 16, 18, 26, and 18 trajectories for 2%, 4%, 6%, and 10% PI(4,5)P_2_ concentrations, respectively, which clearly exhibited left-handed corkscrewing motions, were obtained. To determine the individual corkscrewing pitch, gliding velocity, and rotational velocity, we selected the *xz*-trajectory that showed at least 2 continuous oscillations. The individual corkscrewing pitch was determined by fitting the *xz*-trajectory with a sine function. Individual gliding and rotational velocities were determined by fitting the *t*-*x* and *t*-*rev* trajectories, respectively, with linear functions.

### Statistics and reproducibility

Tracking data for QDs were obtained from at least two independent experiments, and sample sizes are indicated in detail in the text or the “Materials and methods” section.

### Supplementary Information

Supplementary Information 1.

Supplementary Video 1.

Supplementary Information 2.

## Data Availability

All samples used in this study are available from the corresponding authors upon request. The source data for graphs in the main figures are provided as Supplementary Data 1.

## Code availability

Custom scripts for tracking QDs were written using Igor Pro 5.05A. Trajectories were analyzed using MATLAB R2023a. These scripts are available upon reasonable request.
