# Supplementary material for: Membrane-bound myosin IC drives the chiral rotation of the gliding actin filament around its longitudinal axis
Source: Sci Rep. 2023 Nov 14;13:19908. doi: 10.1038/s41598-023-47125-5 (PMC10646037; doi:10.1038/s41598-023-47125-5)
Supplement: Supplementary file 1 — Supplementary Information 1. [file 41598_2023_47125_MOESM1_ESM.pdf]

Supplementary Information for

Membrane-bound myosin IC drives the chiral rotation of the gliding actin filament around its longitudinal axis

Yusei Sato[1#], Kohei Yoshimura[2], Kyohei Matsuda[1], Takeshi Haraguchi[2], Akisato Marumo[1], Masahiko Yamagishi[1], Suguru Sato[2], Kohji Ito[2]* and Junichiro Yajima[1,3,4]*

[1]*Department of Life Sciences, Graduate School of Arts and Sciences, The University of Tokyo, 3-8-1 Komaba, Meguro-ku, Tokyo 153-8902, Japan*

[2]*Department of Biology, Graduate School of Science, Chiba University, 1-33, Inage, Chiba, Japan*

[3]*Komaba Institute for Science, The University of Tokyo, 3-8-1, Komaba, Meguro-ku, Tokyo 153-8902, Japan*

[4]*Research Center for Complex Systems Biology, The University of Tokyo, 3-8-1, Komaba, Meguro-ku, Tokyo 153-8902, Japan*

*Correspondence should be addressed to K.I. (k-ito@faculty.chiba-u.jp) or J.Y. (yajima@bio.c.u-tokyo.ac.jp)

Supplementary figures, tables, and a movie:

Supplementary Figure 1 Diffusivity of lipids in SLBs
Supplementary Figure 2 Tracking uncertainty of 3D measurement using *tPOT* microscope
Supplementary Figure 3 Representative 3D trajectory of a QD bound to a corkscrewing actin filament
Supplementary Figure 4 Dependence of the gliding velocity on myosin IC concentration
Supplementary Figure 5 Purification of *Drosophila* myosin IC
Supplementary Figure 6 Quantification of actin filament length
Supplementary Table 1 Diffusivity of lipids in SLBs
Supplementary Table 2 Corkscrew motion of actin filaments driven by myosin IC on the SLBs
Supplementary Movie 1 Actin filament corkscrewing motion driven by membrane-bound single-headed myosin IC

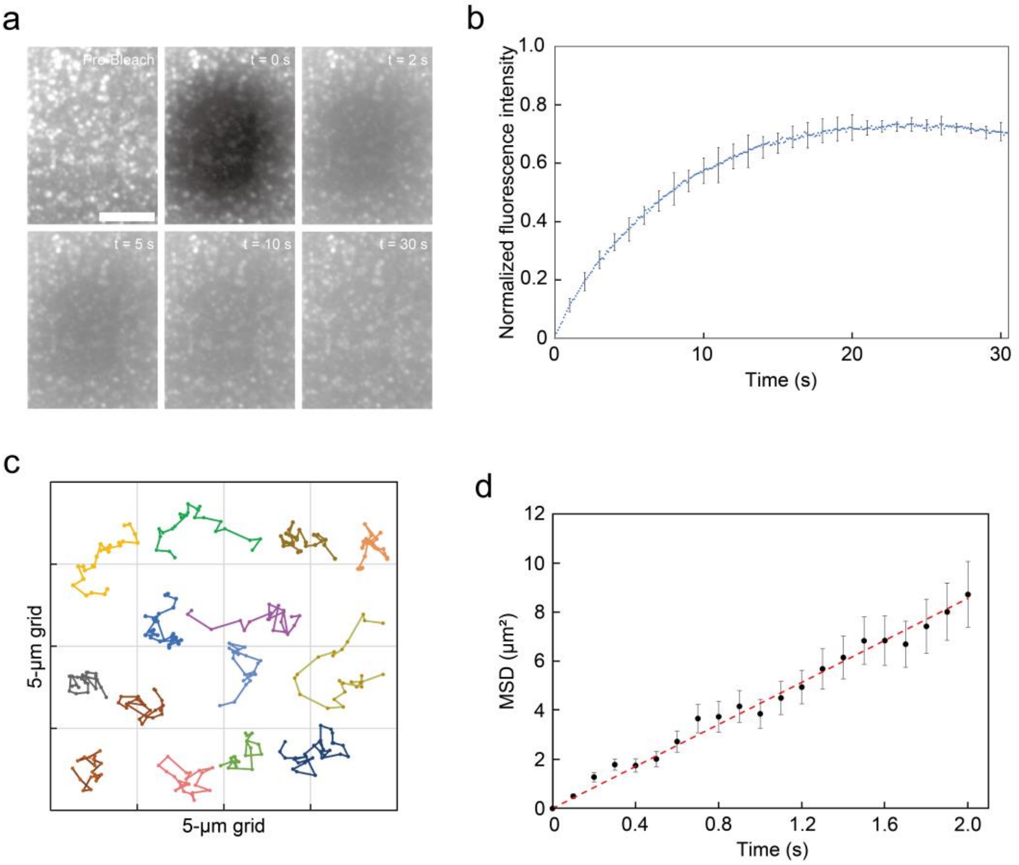

Supplementary Figure 1 | Diffusivity of lipids in SLBs

(**a**) Time-lapse series of FRAP image for an SLB with a molar composition of DOPC: PI(4,5)P_2: ATTO647N–DOPE of 94:6:0.05. Scale bar is 5 μm. (**b**) The average curve of the normalized fluorescence intensity after photobleaching vs. time. The diffusion coefficient of the SLBs was determined to be $D_{\mathrm{LIPID}} = 2.7 \pm 0.4$ μm^2·s^{-1} ($n = 5$, 4 independent SLBs; mean ± SD). (**c**) 14 representative single-molecule trajectories of diffusing Alexa 488-conjugated streptavidin (Avi-488) anchored on a DSPE–PEG-2000–biotin in the SLBs. Biotinylated SLBs were incubated with unlabeled streptavidin (5 nM) and Avi-488 (50 pM). The diffusing single molecules of Avi-488 were excited with a 488 nm laser (488 LS FP, Coherent OBIS) and imaged at 10 frames per seconds using TIRF microscope (IX71, Olympus) equipped with UPlanApo TIRF 100×/1.45 NA objective (Olympus), and an EM-CCD camera (iXon DV887DCS, Andor Technology) via Solis software (Andor Technology). Single particles were tracked using the tracking software Mark2 [1]. (**d**) Mean-square displacement (MSD) plot (black; mean ± SD) of diffusing Avi-488 molecules. The ensemble average diffusion coefficient was obtained to be $D_{\mathrm{Avi}} = 1.1 \pm 0.3$ μm^2·s^{-1} (mean ± SD; $n = 56$ tracked molecules, 11 independent experiments) as characterized by linear fitting to the MSD as function of time.

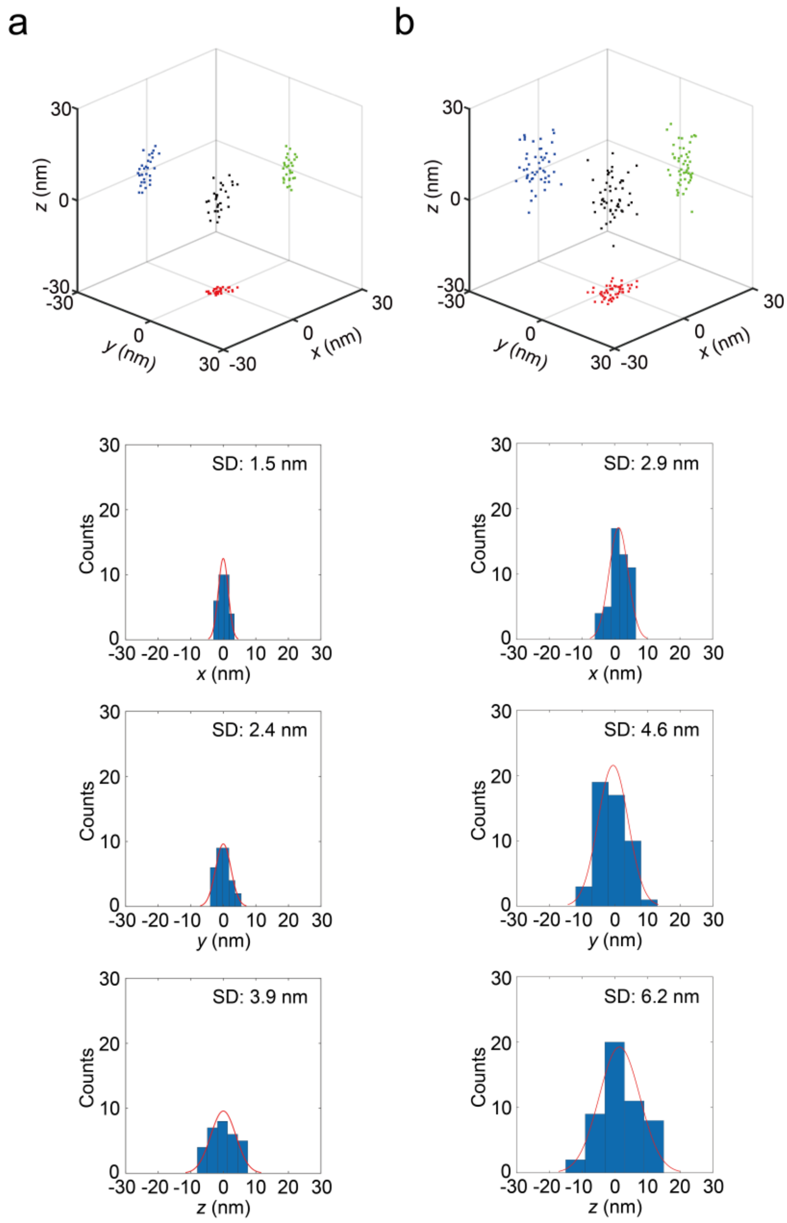

Supplementary Figure 2 | Tracking uncertainty of 3D measurement using *tPOT* microscope

(**Upper**) The 3D plots show the positions of a QD bound to an actin filament fixed on a nitrocellulose-coated glass surface during a 30 second recording at 1 frame per second (**a**) and 2 frames per second (**b**), respectively, obtained using the *tPOT* microscope. *xyz*- (black), *xy*- (red), *xz*- (blue), *yz*- (green) plots are shown. (**Lower**) Histogram of the *x*, *y*, and *z* positions of the QD. The standard deviations (SD) of *x*-, *y*- and *z*-positions were derived from a Gaussian fit. QD-bound actin filaments were infused into the nitrocellulose coated glass chamber and were incubated for 2 min. After incubation, KMg25 buffer containing 1 mM ATP, 3 mg/mL glucose, 50 U/mL glucose oxidase (Sigma-Aldrich), 40 µg/mL catalase (Sigma-Aldrich), 0.3 mg/mL creatin kinase (Roche), 5 mM creatin phosphate (Roche) was infused into the chamber to wash out residual QD-labeled actin filaments, and then the chamber was sealed with vacuum grease.

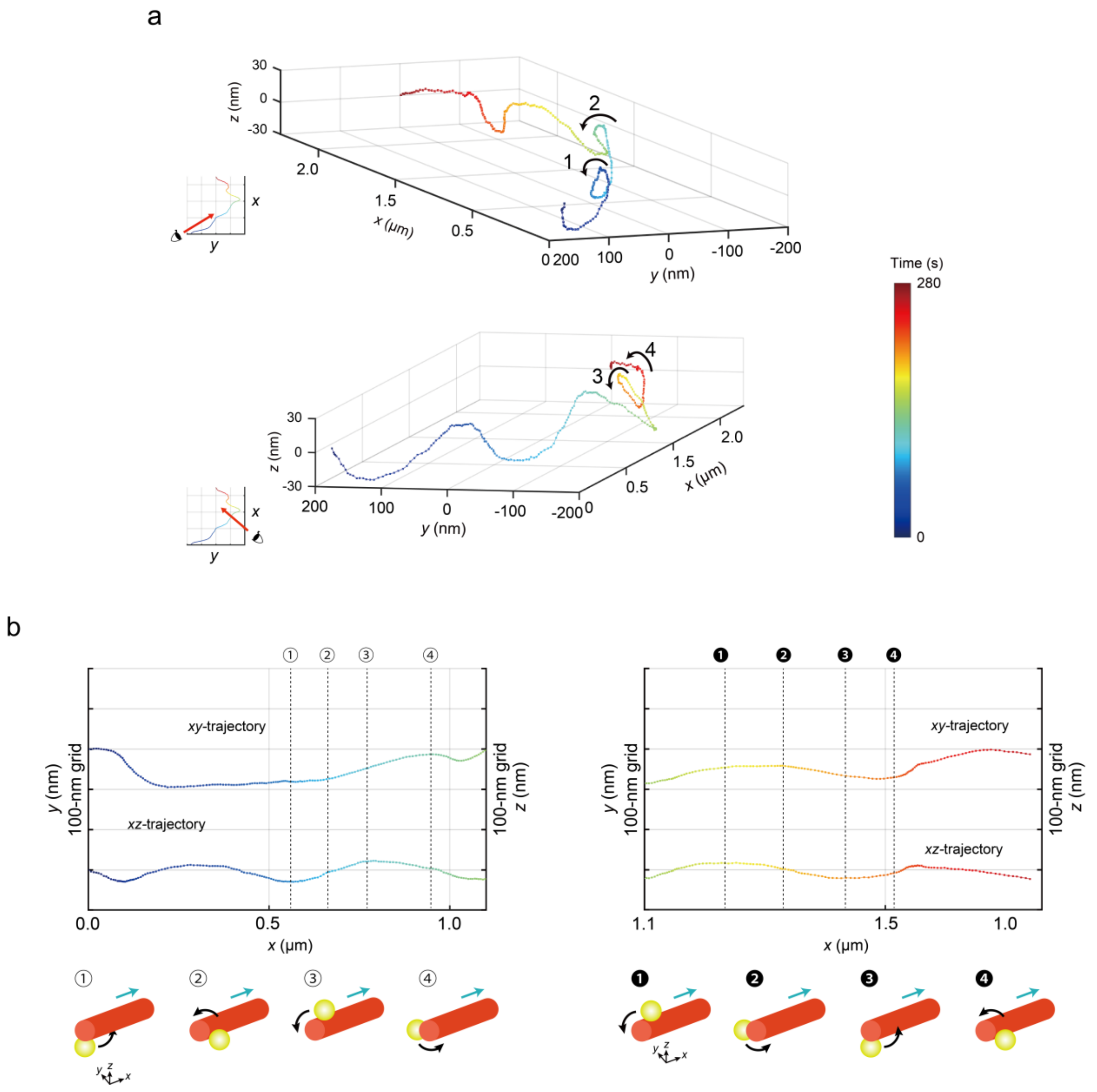

Supplementary Figure 3 | Representative 3D trajectory of a QD bound to a corkscrewing actin filament

(**a**) The 3D plot of a QD-bound actin filament curved in the *x*-*y* direction viewed from different angle. Top: shows the corkscrew motion in the first half of the 3D trajectory; bottom: shows the corkscrew motion in the second half of the 3D trajectory. 3D plot of the QD reveals the left-handed corkscrew motion of the actin filament driven by myosin IC. The color indicates the observation time (see color bar). (**b**) The *xy*- and *xz*-trajectories of the QDs bound to the actin filament shown in **a**. The left and right plot shows first and second halves of the trajectory. Rotation handedness is confirmed by referring to the *xy*- and *xz*-trajectories of the QD, because sinusoidal oscillation phase of the two traces shift with a quarter of the wavelength as the gliding actin filaments rotate around their longitudinal axis. The numbered labels (①–④ and ❶–❹) and thin black dotted lines indicate the peaks and midpoints

for a given period of the sinusoidal-like *xz*-trajectory. Schematic of the left-handed corkscrewing motion of the actin filament over time (bottom). Red cylinder and yellow sphere represent the actin filaments and QDs, respectively. The blue and black curved arrows indicate the gliding an rottion direction of the actin filaments, respectively. The numbers (①–④ and ❶–❹) correspond to those indicated above. Optimally, the *z*- and *y*-coordinates of the QD bound to the actin filament have minimum values at ① and ②, and maximum values at ③ and ④, respectively (bottom left). The *z*- and *y*-coordinates of the QD bound to the actin filament have maximum values at ❶ and ❷, and minimum values at ❸ and ❹ respectively (bottom right).

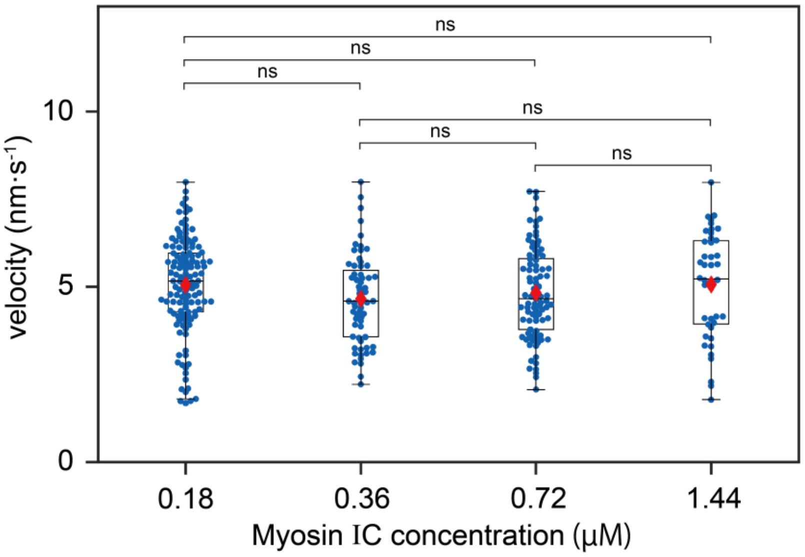

Supplementary Figure 4 | Dependence of the gliding velocity on myosin IC concentration
Gliding velocity of Alexa488-labeled actin filaments without bound QDs was measured on the SLBs containing 6% PI(4,5)P_2 at varying concentrations of Myosin IC. The box represents the 75–25th percentiles, and the median and mean values are indicated by black lines and red diamonds, respectively (*n* = 129 (0.18 μM myosin IC), 63 (0.36 μM), 89 (0.72 μM), 42 (1.44 μM)). ns, nonsignificant in the Welch's t-test. 2 mg/mL of BSA was infused into the biotinylated SLB chamber and incubated for minimum of 30 min for blocking. 40 nM actin filaments (0.5 μL) were mixed with the required volume of 3.25 μM myosin IC in a total of 4.5 μL of KMg25 buffer containing 1 mM ATP, 9.7 μM calmodulin, 3 mg/mL glucose, 50 U/mL glucose oxidase (Sigma-Aldrich), 40 μg/mL catalase (Sigma-Aldrich), 0.3 mg/mL creatine kinase (Roche), and 5 mM creatine phosphate (Roche) immediately before each experiment to prepare the assay mixture. Into the SLB-coated glass flow chamber, one chamber volume of the assay mixture was infused, and the chamber was then sealed using vacuum grease. The assays were performed at 25°C. The gliding velocity was calculated by dividing the distance travelled by the time interval (5 s) using the automated tracking software Mark2[1].

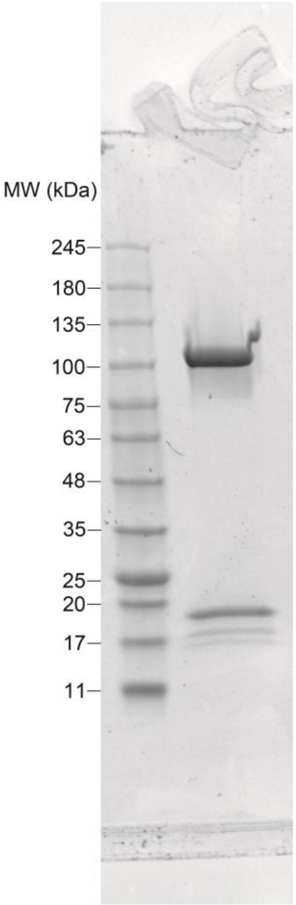

Supplementary Figure 5 | Purification *Drosophila* myosin IC

Representative 4-20% SDS-PAGE gel of purified *Drosophila* myosin IC heavy chain and calmodulin light chain.

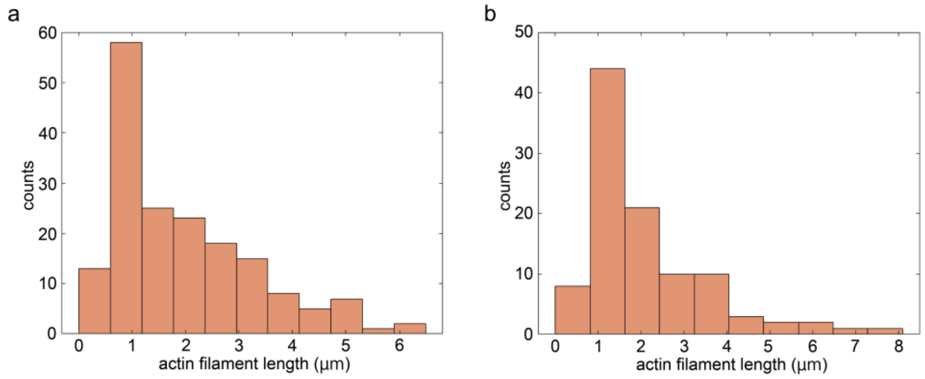

Supplementary Figure 6 | Quantification of actin filament length

Histogram of the length of QD-unbound (**a**) and QD-bound (**b**) actin filaments. The mean lengths of QD-unbound and QD-bound actin filaments were 2.0 ± 1.4 μm (*n* = 175, 3 independent experiments; mean ± SD) and 2.1 ± 1.5 μm (*n* = 102, 5 independent experiments; mean ± SD), respectively. No significant difference in actin filament length by Welch's t-test ($p > 0.5$) were found. The assays were performed under the equally conditions as the corkscrew assay. Actin filaments lengths were measured by determining filament contours using segmented line tool of ImageJ software. Briefly, QD-unbound or QD-bound actin filaments (0.5 μL) were mixed with 1 μL of 3.25 μM myosin-IC in a total of 4.5 μL of KMg25 buffer containing 1 mM ATP, 9.7 μM calmodulin, 3 mg/mL glucose, 50 U/mL glucose oxidase (Sigma-Aldrich), 40 μg/mL catalase (Sigma-Aldrich), 0.3 mg/mL creatin kinase (Roche), and 5 mM creatin phosphate (Roche) immediately before each experiment to prepare the assay mixture. Into the SLB coated glass flow chamber, one chamber volume of the assay mixture was infused and the chamber then sealed using vacuum grease. The assays were performed at 25°C.

Supplementary Table 1 Diffusivity of lipids in SLBs

Lipid	Single particle
D_{LIPID} * (μm^2·s^{-1})	D_{Avi} † (μm^2·s^{-1})
2.7 ± 0.4 (*n* = 5)	1.1 ± 0.3 (*n* = 56)

*Value is mean ± SD (4 independent SLBs)

†Value is mean ± SD (11 independent experiments)

Supplementary Table 2 Corkscrew motion of actin filaments driven by myosin IC on the SLBs

PI(4,5)P_2 concentration	Gliding velocity (nm·s^{-1})	Rotational velocity (rev·s^{-1})	Corkscrew pitch (μm)
2% (*n* = 16)	17.6 ± 4.8	+0.021 ± 0.006	0.84 ± 0.19
4% (*n* = 18)	9.4 ± 2.9	+0.014 ± 0.005	0.69 ± 0.15
6% (*n* = 26)	9.5 ± 4.0	+0.016 ± 0.003	0.58 ± 0.16
10% (*n* = 18)	9.1 ± 2.3	+0.019 ± 0.007	0.50 ± 0.10

p-values resulting from statistical analysis of corkscrew motion

	p-value		
PI(4,5)P_2 concentration pairs	Gliding velocity	Rotational velocity	Corkscrew pitch
2% - 4%	0.00006***	0.00064***	0.02492*
2% - 6%	0.00002***	0.00764**	0.00016***
2% - 10%	0.00002***	0.24075^{ns}	0.00001***
4% - 6%	0.71132^{ns}	0.00927**	0.04495*
4% - 10%	0.60466^{ns}	0.03880*	0.00089***
6% - 10%	0.87646^{ns}	0.28275^{ns}	0.18922^{ns}

*** $p < 0.001$; **$p < 0.01$; * $p < 0.05$; and *ns*, nonsignificant

Supporting Movie 1

The QD bound to corkscrewing actin filament driven by SLB-bound myosin IC molecules The movie shows Alexa 647 labeled gliding actin filament obtained for the first ~3 seconds (1.0 s intervals, ×1 actual speed, Cy5 filter set, Semrock), and a QD bound to the gliding actin filament for the remaining ~7 seconds (1.0 intervals, ×100 actual speed, GFP-3035C filter set, Semrock). Scale bar = 5 μm. The image is 19.4 μm high and 33.8 μm wide.

Reference for supplementary information

1. Furuta, K. & Toyoshima, Y. Y. Minus-End-Directed Motor Ncd Exhibits Processive Movement that Is Enhanced by Microtubule Bundling In Vitro. *Curr. Biol.* **18**, 152–157 (2008).
